# Helminth Parasites of Invasive Freshwater Fish in Lithuania

**DOI:** 10.3390/ani14223293

**Published:** 2024-11-15

**Authors:** Olena Kudlai, Vytautas Rakauskas, Nathan Jay Baker, Camila Pantoja, Olga Lisitsyna, Rasa Binkienė

**Affiliations:** 1 Nature Research Centre, 08412 Vilnius, Lithuania; nathan93baker@gmail.com (N.J.B.); rasa.binkiene@gamtc.lt (R.B.); 2 Institute of Parasitology, Biology Centre of the Czech Academy of Sciences, 37005 České Budějovice, Czech Republic; camilaspantoja@yahoo.com.br; 3 I.I. Schmalhausen Institute of Zoology, NAS of Ukraine, 01030 Kyiv, Ukraine; olisitsyna@izan.kiev.ua

**Keywords:** aquatic invasion, *Neogobius* spp., *Perccottus glenii*, *Pseudorasbora parva*, morphological and molecular identification, Baltic freshwater ecosystems

## Abstract

Freshwater ecosystems in Lithuania are threatened by the introduction of invasive fish species including *Neogobius fluviatilis*, *N. melanostomus*, *Perccottus glenii*, and *Pseudorasbora parva*. Data on helminth parasites of these fishes have not been comprehensively studied, with only two reports on parasites of *N. melanostomus* from the Curonian Lagoon and Baltic Sea, Lithuania. We examined 278 fish individuals representing 4 invasive species from 13 waterbodies. Using morphological and molecular analyses, we identified 29 helminth taxa representing 15 digenean trematodes, 6 nematodes, 4 cestodes, 2 acanthocephalans, and 2 monogeneans. With 18 species, *N. fluviatilis* had the highest helminth diversity, followed by *N. melanostomus* (11 species) and *Ps. parva* (8 species). *Perccottus glenii* was found to be free from helminth infection. The availability of historical information on the native fish parasites in Lithuania allowed us to conclude that out of the 29 recorded species, invasive fish serve as hosts for 22 local fish helminth species, while 7 helminth species have been reported exclusively in invasive fish. Based on newly obtained and previously published data, a total of 34 helminth species parasitise invasive fish in Lithuania, of which 30 use these fish as intermediate or paratenic hosts.

## Simple Summary

In this paper, we studied the parasitic worms of invasive fish in Lithuania. We focused on four fish species foreign to Lithuanian freshwaters, namely the monkey goby (*Neogobius fluviatilis*), the round goby (*Neogobius melanostomus*), the stone moroko (*Pseudorasbora parva*), and the Chinese sleeper (*Perccottus glenii*). In total, we examined 278 fish from 13 freshwater bodies and found 29 species of parasitic worms. Most of them were found to be juvenile and we used morphological and DNA-based methods to identify these parasitic worms. The monkey goby was the most infected fish species, carrying 18 species of parasitic worms, while the Chinese sleeper was, surprisingly, not infected. Most of the parasitic worms we found were already present in fish in Lithuania, but seven were only found within the invasive fish species we examined. This means that these fish likely brought some parasites with them when they were first introduced to Lithuanian freshwaters and are also capable of carrying and spreading local Lithuanian parasites. This novel information is important, as it helps us to better understand how invasive fish might be affecting ecosystems beyond the well-known effects that they have on habitats and other local animals.

## 1. Introduction

The invasion and proliferation of non-native species is considered one of the main threats to global biodiversity, leading to extinctions and population declines that have been reported across various ecosystems [1,2]. Notably, freshwater environments are among the most invaded and threatened ecosystems worldwide [3,4], despite harbouring a disproportionate amount of global vertebrate and invertebrate biodiversity [5]. While the various processes facilitating the spread of economically important non-native vertebrate taxa such as fish are well known [6,7], including, for example, shipping, morphological alterations (i.e., canals), and the aquarium trade, much less is known about the spread of non-native parasitic invertebrate species, which can co-invade with their fish hosts [8,9]. Accordingly, our understanding of non-native parasite diversity is far from complete, particularly in regions of the world that are comparatively underrepresented in the literature.

Lithuania is one such region that is enriched with freshwater ecosystems of different types, some of which are affected and/or threatened by the invasion of non-native species, including fish [10]. Over the past three decades, a rapid expansion of Ponto–Caspian gobies has been observed in the rivers connected to the central European invasion corridor [11,12]. The Nemunas River catchment, which is connected to the Pripyat, Vistula, and Berezina rivers by canals, forms connections between the watersheds of the Nemunas and Dnieper rivers. Such connections make this the most probable pathway for new invasions in this region, primarily those of Ponto–Caspian origin [13]. Currently, two gobies of Ponto–Caspian origin, the monkey goby *Neogobius fluviatilis* (Pallas, 1814) and the round goby *N. melanostomus* (Pallas, 1814), are reported to be present in the Nemunas River basin [14,15]. *Neogobius melanostomus* was recorded in the Nemunas River basin for the first time in 2002 [16]. However, the distribution of *N. melanostomus* remains restricted to the Curonian Lagoon and the outlets of the River Nemunas [10]. *Neogobius melanostomus* has never been reported from other parts of the Nemunas River basin [15,17], suggesting that it has naturally entered the Nemunas River drainage area from the Baltic Sea via the Curonian Lagoon. Meanwhile, initial reports of *N. fluviatilis* in the Nemunas River drainage basin date back to 2013 [14] and it has been annually observed in the Nemunas River basin in Belarus since 2013 and in Lithuania since 2015 [15].

In addition to the two gobiid species, there are two other invasive fish species of European concern present in Lithuania, namely the Chinese sleeper *Perccottus glenii* (Dybowski, 1877) and the stone moroko *Pseudorasbora parva* (Temmick et Schlegel, 1846). Although both species occupy similar habitats (small, eutrophic waterbodies overgrown by water plants and with degraded fish assemblages), the current introduction vectors and pathways of these species, as well as their impact on local freshwater fish, are different [18].

It is well documented that parasites, specifically helminths, co-invade ecosystems outside of their native range along with their non-native fish hosts [8]. The non-native hosts introduce parasites from their native range and transmit parasites to native species (spillover) or acquire parasites from native species (spillback), increasing the abundance of the native parasite species and the likelihood of native hosts becoming infected [19]. To fully understand the consequences of these alterations and the impact of invasion on the transmission of local parasites and the composition and dynamics of local parasite communities, the assessment of parasites in invasive fish species is essential, with accurate parasite identification being a prerequisite. Considering that many helminth parasites use freshwater fish as their intermediate hosts, they are mainly found at their larval stages, often having simple morphology and lacking reliable features for identification. Moreover, helminth parasites at this stage are often less host-specific [20,21]. Thus, morphology-based identification should be verified using DNA sequence analysis.

To date, only two attempts have been made to address the species diversity of parasites of *N. melanostomus* in Lithuania [16,22], with the authors reporting on a total of seven helminth species, while data on parasites of the other three invasive species are absent. By applying an integrative taxonomic approach to the identification of helminth parasites from invasive fish species in Lithuania, this study aimed to contribute to our understanding of the diversity of helminths in *N. fluviatilis*, *N. melanostomus*, *Pe. glenii,* and *Ps. parva*, including their infection rates and distribution. We compare this newly obtained data to data from other regions in which these fish species are invasive. Since the River Nemunas represents one of the main aquatic invasion corridors in Europe, these data will allow us to study parasite dynamics at the important juncture between invasive fish species introduction and establishment.

## 2. Materials and Methods

### 2.1. Study Area

Lithuania belongs to the Baltic Sea drainage basin, is situated along the south-eastern shore of the Baltic Sea, and has a territory of approximately 64,800 km^2^, which is transected by seven main river basins [23].

**Figure 1:**
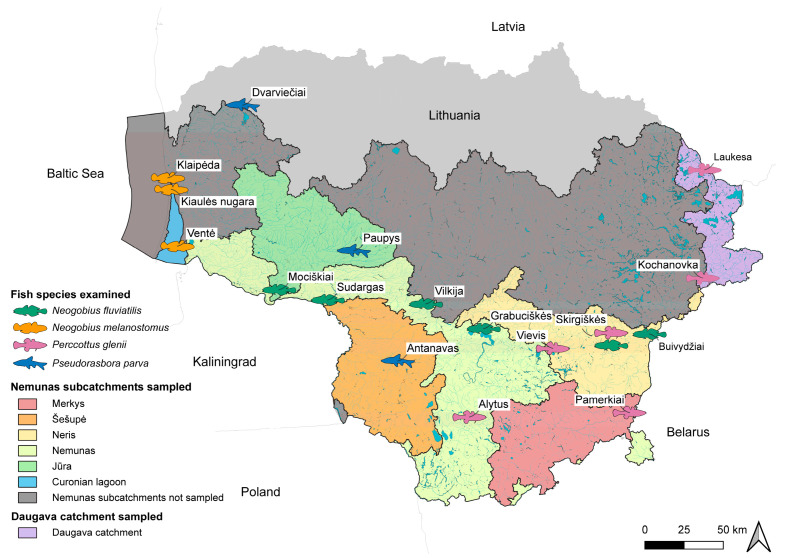
Map showing sampling sites in Lithuania from where fish were collected (coordinates of localities are in Table 1).

The River Nemunas has the largest catchment area in Lithuania, with 93% of Lithuanian territory being within or connected by canals to the Nemunas River basin. Of note is that the Nemunas River drainage basin is connected to the Pripyat, Vistula, and Berezina rivers by canals forming a northern branch of the central European invasion corridor [15,24]. Invasive fish species for parasitological assessment were sampled in both the Nemunas and Daugava river basins within the territory of Lithuania (Figure 1).

### 2.2. Fish Sampling

Four invasive fish species were examined in the present study, namely *N. fluviatilis* (n = 125) and *N. melanostomus* (n = 36) (Gobiidae), *Pe. glenii* (Odontobutidae) (n = 55), and *Ps. parva* (Gobionidae) (n = 62). Specimens of *N. melanostomus* and *N. fluviatilis* were collected from nine sites represented by both lentic and lotic habitats within the Nemunas River basin (Figure 1, Table 1). Specimens of *Pe. glenii* were collected from four lentic and two lotic sites within the Nemunas and Daugava river basins, while specimens of *Ps. parva* were collected from one lentic and two lotic sites within the Nemunas River basin (Figure 1, Table 1). Fish were sampled in 2022 and 2023 from May until September. Specimens of *N. fluviatilis* and *Ps. parva* were collected using battery-powered electric fishing gear (Samus Special Electronics, Samus-725 mp; Hans Grassl GmbH, Schönau am Königssee, Germany), while *N. melanostomus* were collected using baited crayfish traps (50 cm width and 80 cm length) or beach seine (7 m length, 4 mm mesh size) depending on habitat conditions. Specimens of *Pe. glenii* from lentic environments were collected with multi-mesh benthic gillnets, each of which was 40 m in length and 3 m in height. Mesh size varied every 5 m and was 14, 18, 22, 25, 30, 40, 50, and 60 mm. Specimens of *Pe. glenii* from lotic habitats were captured using electric fishing gear. Pursuant to state fishing laws and sampling licensing, all native fish species individuals were released.

Sampling was carried out under the permits obtained from the Environment Protection Agency, Lithuania (license No.: 018, 019 valid for the 2022 year; 016, 017 valid for the 2023 year). The collected fish were transported live to a laboratory aquarium, where they were dissected within several days after sampling. In total, 278 specimens were examined, measured to the nearest 1 mm, weighed to the nearest 0.1 g, and their age was determined from scales [25] prior to dissection (Table 1). Fish were identified using the identification key provided by Kottelat and Freyhof [26] and taxonomy followed FishBase [27].

### 2.3. Parasitological Examination and Sample Collection

Fish were individually labelled and examined for helminth parasites according to a standardised necropsy protocol; fins, body surface, gills, muscles, and all internal organs were separately examined. Examination was carried out with the aid of high magnification dissecting microscopes Nikon SMZ445 and Nikon SMZ445T (Tokyo, Japan). After detecting and collecting helminth specimens, all fish organs were pressed individually between Petri dishes and screened for additional specimens and species. All helminths were collected, rinsed in saline solution, counted, cold- or heat-killed in saline solution, and preserved in 80% ethanol. The morphology of helminths selected for molecular analyses was initially studied live and a series of photomicrographs were made for each isolate (photogenophores) using a digital camera AxioCam ERc5s on Zeiss Primo Star light microscope (Oberkochen, Germany). These specimens were then transferred to 96% molecular-grade ethanol. Identification of the collected helminths to the lowest possible taxonomic level was achieved via morphological and molecular analyses. Solely morphological analysis was applied in cases when DNA extractions were unsuccessful. The morphology of acanthocephalans and nematodes was studied on temporary total mounts cleared in Berlese’s medium and glycerine, respectively. The material used in this study is stored in the helminthological collection of Nature Research Centre, Lithuania. The parasitological indices (prevalence, P %, and intensity, presented as min–max) were calculated according to Bush et al. [28].

### 2.4. Generation of Sequence Data and Phylogenetic Analyses

Photogenophores and additional specimens intended for molecular identification were used for DNA extraction by applying the KAPA Express Extract Kit (KAPA Biosystems, Cape Town, South Africa) following the manufacturer’s protocols. Depending on the taxonomic group of the studied helminths, different genetic markers were used for the generation of the partial 18S and 28S rRNA, partial mitochondrial cytochrome *c* oxidase subunit 1 (*cox*1) and complete ITS2 sequences. Primer combinations and PCR protocols used for individual gene fragment amplifications are presented in Table 2.

Amplified DNA was purified through the ExoSAP-IT PCR Cleanup enzymatic kit from Thermo Fisher Scientific, Inc. (Waltham, MA, USA) and sequenced from both strands using the PCR primers. Geneious Prime software ver. 2023.2.5 (Biomatters, Auckland, New Zealand) was used to assemble and revise newly obtained sequences. The novel sequences were deposited in GenBank (Supplementary Table S1).

The taxonomic identity of the isolates was checked using the Basic Local Alignment Search Tool (BLAST) (www.ncbi.nlm.nih.gov/BLAST/ accessed on 6 July 2024), and thereafter aligned with the selected representative sequences from GenBank for the phylogenetic analyses. A total of 12 alignments (7 28S rDNA alignments for cestodes, digeneans, and monogeneans, and 5 *cox*1 alignments for digeneans) were built using MUSCLE implemented in Geneious Prime and used for analyses. Alignment 1 (*Archigetes* spp., 1240 nucleotides (nt)), Alignment 2 (*Paradilepis* spp., 1509 nt), Alignment 3 (representatives of the Lytocestidae, 1302 nt), and Alignment 4 (*Eubothrium* spp., 1268 nt) all consisted of the 28S rDNA sequences of cestodes. Two alignments, Alignment 5 (405 nt) and Alignment 6 (1041 nt), consisted of *cox*1 and 28S rDNA sequences of the representatives of the Bucephalidae; Alignment 7 (351 nt) and Alignment 8 (1121 nt) consisted of *cox*1 and 28S rDNA sequences of the representatives of the Cyathocotylidae. Three alignments, Alignment 9 (402 nt), Alignment 10 (360 nt), and Alignment 11 (393 nt), consisted of the *cox*1 sequences of the representatives of the families Diplostomidae, Heterophyidae and Strigeidae, respectively. Finally, Alignment 12 (*Dactylogyrus* spp., 735 nt) consisted of the 28S rDNA sequences of monogeneans. The *cox*1 alignments were aligned with reference to the amino acid translation, using the trematode mitochondrial code (transl_table=21; https://www.ncbi.nlm.nih.gov/Taxonomy/Utils/wprintgc.cgi#SG21 accessed on 6 July 2024) [39,40].

Bayesian inference (BI) analyses were carried out using MrBayes software ver. 3.2.3 [41] and maximum likelihood (ML) analyses were carried out using PhyML ver. 3.0 [42], run on the ATGC bioinformatics platform (http://www.atgc-montpellier.fr/ accessed on 6 July 2024).

The best-fitting model for each analysis was estimated with jModelTest 2.1.2 [43]. These were the GTR + I + G (Alignments 2, 6, 8, 11 and 12), GTR + G (Alignments 3, 4, 5, and 10), HKY (Alignment 1), HKY + I + G (Alignment 7), and HKY + G (Alignment 9). In BI analysis, Markov Chain Monte Carlo simulations were run for 3,000,000 generations, log-likelihood scores were recorded to estimate burn-in, and only the last 75% of trees were used to build the consensus tree. In ML analyses, a nonparametric bootstrap validation was based on 100 pseudoreplicates. FigTree software ver. 1.4 [44] was used to visualise the obtained phylogenetic trees.

Following the nomenclature of previously published studies that provided numbers for unidentified species of *Apatemon* [45,46,47,48] and *Cyathocotyle* [49], we gave the subsequent numbers for the species found in our study: *Apatemon* sp. 7 and *Apatemon* sp. 8, and *Cyathocotyle* sp. 3 and *Cyathocotyle* sp. 4. Despite the updates in the classification of large taxomonic groups of helminth parasites, we refer to helminths recorded in this study as acanthocephalans, cestodes, digenean trematodes, monogeneans and nematodes. 

## 3. Results

Out of the 278 examined fish individuals belonging to 4 species, 173 (62%) were infected with at least 1 species of helminth (Table 1). A total of 78,027 helminth individuals were recorded. Using a combination of morphological and molecular techniques, 29 helminth species belonging to the 5 large taxonomic groups, Acanthocephala (2), Cestoda (4), Digenea (15), Monogenea (2), and Nematoda (6) were identified. Of these, the highest number of species was recorded in *N. fluviatilis* (18), followed by *N. melanostomus* (11) and *Ps. parva* (8). All examined *Pe. glenii* individuals from all sampling sites were not infected with helminths.

Barring monogeneans and one species of nematode, *Pseudocapillaria tomentosa* (Dujardin, 1843), all other helminths were found at the larval stages. Since species identification of larval stages is difficult due to their simple morphology at this early stage of development, DNA-based analyses were used for the identification of collected helminths to the lowest possible taxonomic level. A total of 115 novel sequences (78 of *cox*1 mtDNA, 34 of 28S rDNA, 2 of ITS2 region, and 1 of 18S rDNA) were generated for 103 isolates in this study (Supplementary Table S1).

**Phylum Acanthocephala**
**Class Palaeacanthocephala**

Two species of acanthocephalans were found to parasitise *Neogobius* spp. in the present study. Cystacanths of ***Acanthocephalus anguillae*** (Müller, 1780) (Echinorhynchidae) were found attached to the intestine or in liver of the monkey goby *N. fluviatilis* from the Neris (Skirgiškės, P = 11%, 1 specimen per fish) and Nemunas (Vilkija, P = 9%, 1) rivers. The identification of this species was based solely on morphological data. *Acanthocephalus anguillae* is a common parasite of the digestive tract of many freshwater fish and has been reported from Europe, Asia, and North America [50]. Our record represents the first finding of this species in *N. fluviatilis*, suggesting that this fish can serve as a paratenic host. *Acanthocephalus anguillae* was previously reported in *N. melanostomus* from the Curonian Lagoon, Lithuania [16], from the Baltic Sea near Palanga, Lithuania [22], and from the River Dyje, within the Danube River basin, Czechia [51]. Neither *N. melanostomus* nor *N. fluviatilis* have been reported as hosts for *A. anguillae* in their native distribution ranges.

**Figure 2:**
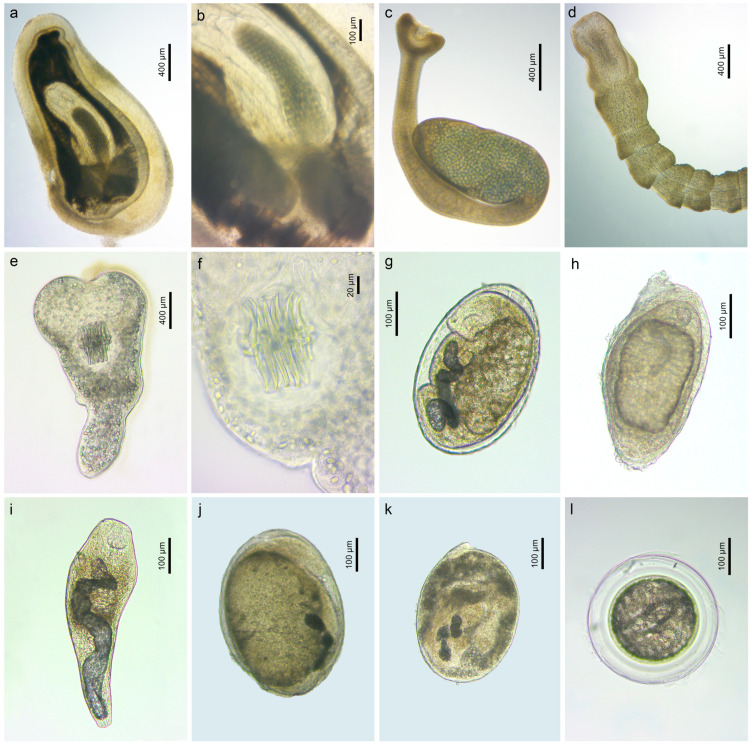
Microphotographs of the helminth parasites found in invasive fish in the present study: (**a**,**b**) *Corynosoma semerme*, live ex *N. melanostomus* (voucher, PQ570008, PQ557260); (**c**) *Archigetes sieboldi*, live ex *N. melanostomus* (voucher PQ570009); (**d**) *Eubothrium crassum*, live ex *N. melanostomus* (voucher, PQ570012); (**e**,**f**) *Paradilepis scolecina*, live ex *N. melanostomus* (voucher, PQ570010); (**g**) *Bucephalus polymorphus*, live, encysted ex *N. fluviatilis* (voucher, PQ582078, PQ560767); (**h**) *B. polymorphus*, live, encysted ex *N. fluviatilis* (voucher, PQ560768); (**i**) *B. polymorphus*, live, excysted ex *N. fluviatilis* (voucher, PQ560766); (**j**) *Rhipidocotyle campanula*, live ex *Ps. parva* (voucher, PQ560769); (**k**) *Rhi. fennica*, live ex *Ps. parva* (voucher, PQ560773); (**l**) *Cyathocotyle prussica* ex *N. melanostomus* (voucher, PQ560792).

A single cystacanth of ***Corynosoma semerme*** (Forssell, 1904) (Polymorphidae) (Figure 2a,b) was found in the liver of a round goby *N. melanostomus* from the Curonian Lagoon (P = 5%). The identification of this specimen was based on both morphology and DNA sequences (18S and 28S rDNA) via comparison with sequence data available in GenBank. This is the first report of *Co. semerme* parasitising species of *Neogobius* in Lithuania. However, *Co. semerme* was recently reported in round goby from the Baltic Sea in Estonia [52]. This species does not parasitise the here studied gobiid species in their native distribution ranges. Adults of *Co. semerme* are common parasites of marine mammals and were previously found in grey and ringed seals in the Baltic Sea [53]. Prior to our study, three species of acanthocephalans, *A. anguillae*, *Echinorhynchus gadi* Zoega in Müller, 1776, and *Pomphorhynchus laevis* (Zoega in Müller, 1776), were exclusively reported in the round goby in Lithuania [16,22].

**Phylum Platyhelminthes**
**Class Cestoda**
**Subclass Eucestoda**

**Figure 3:**
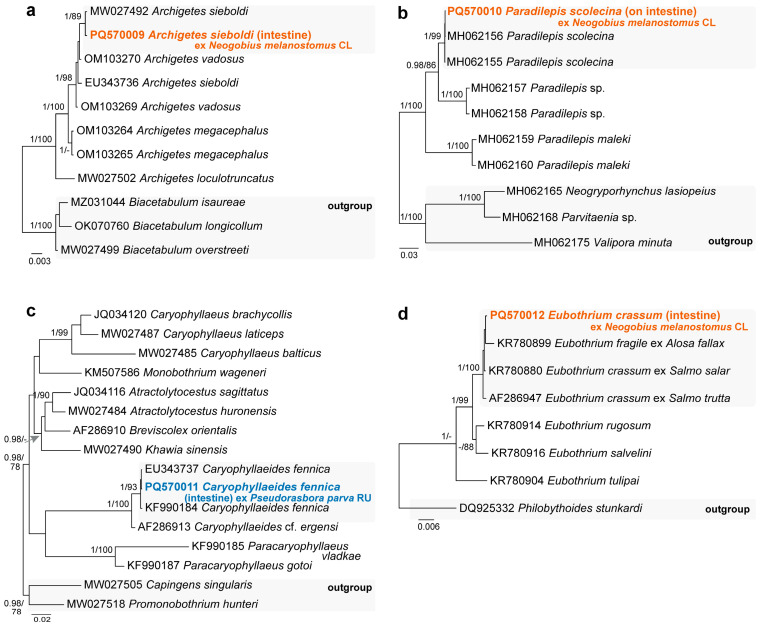
Phylograms resulting from Bayesian inference (BI) analyses based on the 28S rDNA sequences of (**a**) *Archigetes* spp., (**b**) *Paradilepis* spp., (**c**) representatives of the Lytocestidae, and (**d**) *Eubothrium* spp. with nodal support values shown at the node as BI/ML (maximum likelihood). Only values > 0.90 (BI) and >70 (ML) are displayed. Scale bar indicates the expected number of substitutions per site. Sequences generated in this study are in bold and the colour corresponds to the colour of their fish host (see Figure 1, Table 1 for abbreviations).

In the present study, cestodes belonging to four species were recorded; all cestode species were identified based on the 28S rDNA sequence analyses (Figure 3). Two individuals of ***Archigetes sieboldi*** Leuckart, 1878 (Caryophyllidea) (Figure 2c) and one individual of ***Eubothrium crassum*** (Bloch, 1779) (Triaenophoridae) (Figure 2d) were found in the intestine of *N. melanostomus* in the Curonian Lagoon (Klaipėda) with a prevalence of 5% for both species. A single individual of ***Caryophyllaeides fennica*** (Schneider, 1902) (Lytocestidae) infecting the intestine of *Ps. parva* from the River Upė was recorded (P = 6%). ***Paradilepis scolecina*** (Rudolphi, 1819) (Gryporhynchidae) (Figure 2e,f) was recorded in the intestinal wall of both *N. melanostomus* from the Curonian Lagoon (Klaipėda, P = 5%) and *Ps. parva* from the River Upė (P = 6%).

Out of the four cestode species, *Eub. crassum* is known to parasitise *N. melanostomus* in the Vistula Lagoon (Poland), while *Pa. scolecina* is known to parasitise *N. fluviatilis* and *N. melanostomus* in the Vistula Lagoon (Poland) and the River Rhine (Germany) [51]. Both, *Eub. crassum* and *Pa. scolecina* parasitise monkey and round gobies in their native distributions [51]. *Eubothrium crassum* is a parasite of salmonid fish in the northern part of Europe [54], while *Pa. scolecina* is a cosmopolitan parasite of fish-eating birds, mainly cormorants and pelicans [55]. Cestodes *Pa. scolecina* were reported from *Phalacrocorax carbo sinensis* (Linnaeus, 1758) in the Curonian Lagoon [56].

Our study is the first to report cestodes *Ar. sieboldi* and *Ca. fennica* parasitising *N. melanostomus* and *Ps. parva*, respectively. *Caryophyllaeides fennica* infects a wide spectrum of distantly related cyprinid fishes, whereas *Ar. sieboldi* is primarily a parasite of oligochaetes. Both species occur in the Palaearctic and Nearctic regions [57,58].

**Class Trematoda**
**Subclass Digenea**

With 15 recorded species, digenean trematodes were the most diverse helminth group in the present study. The recorded species are represented by the five families, namely Bucephalidae, Cyathocotylidae, Diplostomidae, Heterophyidae, and Strigeidae.

**Figure 4:**
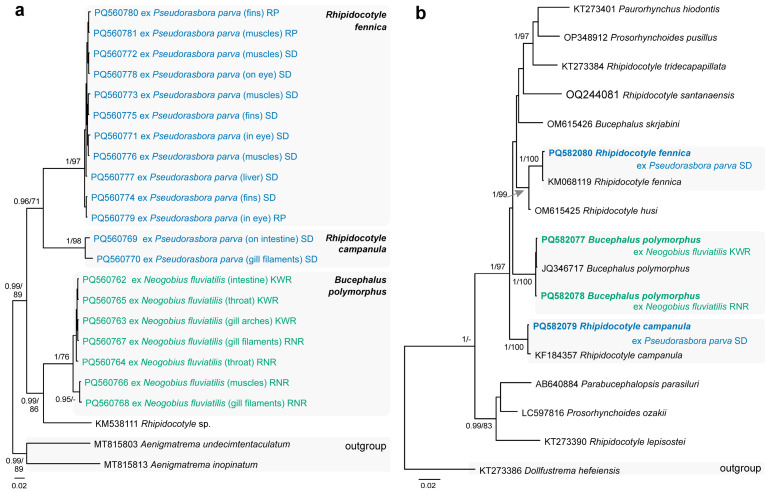
Phylograms resulting from Bayesian inference (BI) analyses based on the (**a**) *cox*1 mtDNA and (**b**) 28S rDNA sequences of the members of the Bucephalidae with nodal support values shown at the node as BI/ML (maximum likelihood). Only values > 0.90 (BI) and >70 (ML) are displayed. Scale bar indicates the expected number of substitutions per site. Sequences generated in this study are in bold and the colour corresponds to the colour of their fish host (see Figure 1, Table 1 for abbreviations).

The bucephalid species, ***Bucephalus polymorphus*** von Baer, 1827, was found encysted and excysted (Figure 2g–i) in/on a variety of host tissues (gills, throat muscles, intestine, liver, and muscles) in *N. fluviatilis* from the Kaunas Water Reservoir (P = 30%, 1–7) as well as from the Nemunas (Vilkija, P = 6%, 1) and Neris [Buivydžiai (P = 20%, 1–4) and Skirgiškės (P = 30%, 1–44)] rivers. The identification of this species, especially when found as encysted metacercariae (Figure 2g,h), was based on the *cox*1 and 28S rDNA sequence analyses (Figure 4). Prior to our study, *B. polymorphus* was reported from the round goby in the Danube (Austria), Dnieper (Ukraine), Morava, and Dyje (Czechia) rivers and the Stettiner Haff (Germany). It was also reported in the monkey goby from the Bugo-Narew (Vistula basin) and Włocławek reservoirs, the River Vistula (Poland), and the River Rhine (Germany) [51]. *Bucephalus polymorphus* is a parasite infecting the intestines of predatory fish (Percidae, Esocidae, etc.) and is known from both monkey and round gobies in their native distribution ranges [51].

Two other members of this family, ***Rhipidocotyle campanula*** (Dujardin, 1845) (Figure 2j) and ***Rhi. fennica*** Gibson, Taskinen & Valtonen, 1992 (Figure 2k) were found in *Ps. parva*. Metacercariae of *Rhi. campanula* were found on the intestine and gills filaments in fish collected from a swamp near Dvarviečiai (P = 6%, 1–2) and metacercariae of *Rhi. fennica* were found in different host tissues (in the eyes, intestinal wall, liver, muscles, and fins) in fish from a swamp near Dvarviečiai (P = 82%, 1–76) and the River Pilvė (P = 82%, 1–73). Species delimitation and identification of both species of *Rhipidocotyle* (Figure 2j,k) was based on the 28S rDNA and *cox*1 sequence analyses (Figure 4). Our study is the first to report both species parasitising *Ps. parva*.

**Figure 5:**
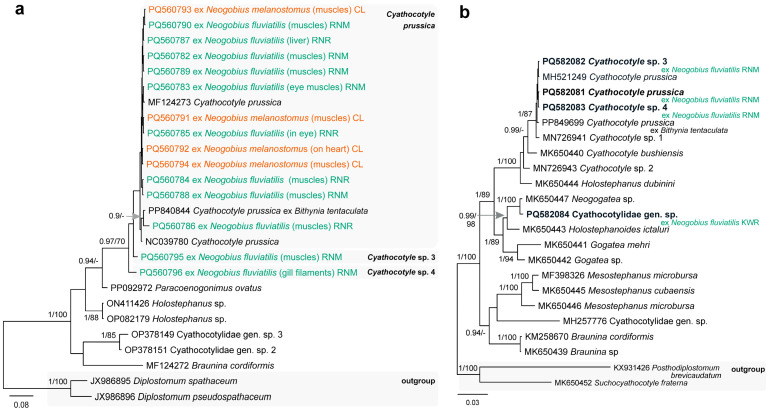
Phylograms resulting from Bayesian inference (BI) analysis based on the (**a**) *cox*1 mtDNA and (**b**) 28S rDNA sequences of the members of the Cyathocotylidae with nodal support values shown at the node as BI/ML (maximum likelihood). Only values > 0.90 (BI) and >70 (ML) are displayed. Scale bar indicates the expected number of substitutions per site. Sequences generated in this study are in bold and the colour corresponds to the colour of their fish host (see Figure 1, Table 1 for abbreviations).

The Cyathocotylidae was represented by four species, with a single species identified at the species level. Due to the similar morphology of the metacercariae, species delimitation and identification was based solely on the 28S rDNA and *cox*1 mtDNA analyses (Figure 5). Metacercariae of ***Cyathocotyle prussica*** Mühling, 1896 (Figure 2l) were found in the gills, eyes, and muscles, and on the heart and liver of *N. fluviatilis* and *N. melanostomus*, whereas the other species, ***Cyathocotyle* sp. 3**, ***Cyathocotyle* sp. 4** and **Cyathocotylidae gen. sp.**, were found in gills, eyes, and muscles, and on the heart and fins of *N. fluviatilis*. Individuals of *N. fluviatilis* were infected with cyathocotylid metacercariae from the Kaunas Water Reservoir (10%, 1–2), as well as from the Jūra (P = 39%; 1–35), Nemunas [Sudargas (P = 1 out of 1, 3) and Vilkija (P = 41%, 1–21)] and Neris [Buivydžiai (33%, 1–2) and Skirgiškės (P = 52%, 1–9)] rivers, whereas individuals of *N. melanostomus* were infected from the Curonian Lagoon [Ventė (P = 60%, 1–11) and Klaipėda (P = 5%; 1–19)]. Metacercariae of *C. prussica* have been reported from monkey gobies in the River Vistula (Poland) [51] and have not been reported from round or monkey gobies in their native distribution ranges. Adults of the genus *Cyathocotyle* are parasitic in birds, whereas adults of other members of the Cyathocotylidae parasitise fish, reptiles, birds, and mammals [59].

**Figure 6:**
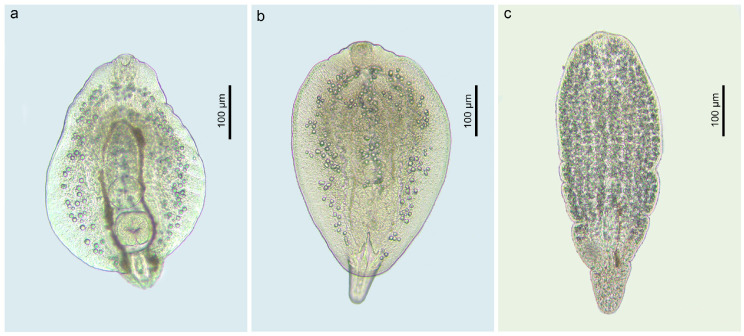
Microphotographs of the helminth parasites found in invasive fish in the present study: (**a**) *Diplostomum pseudospathaceum*, live ex *N. fluviatilis* (voucher, PQ560800); (**b**) *Di. spathaceum*, live ex *N. melanostomus* (voucher, PQ560815); (**c**) *Tylodelphys clavata*, live ex *N. melanostomus* (voucher, PQ560825).

**Figure 7:**
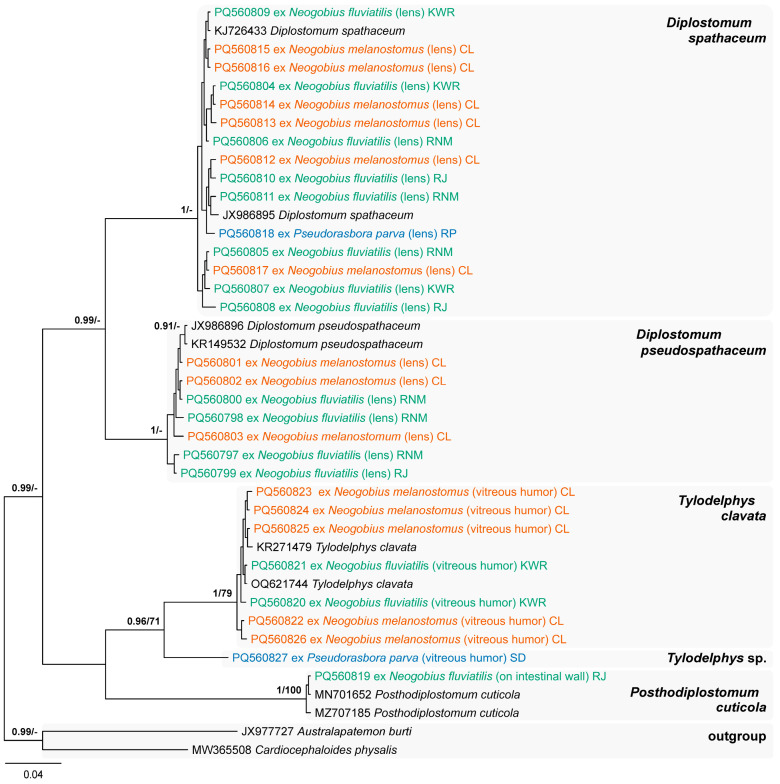
Phylogram resulting from Bayesian inference (BI) analysis based on the *cox*1 mtDNA sequences of the members of the Diplostomidae with nodal support values shown at the node as BI/ML (maximum likelihood). Only values > 0.90 (BI) and >70 (ML) are displayed. Scale bar indicates the expected number of substitutions per site. Sequences generated in this study are in the colour corresponding to the colour of their fish host (see Figure 1, Table 1 for abbreviations).

The most diverse family of digenean trematodes infecting invasive species in our study was the Diplostomidae, with five species recorded, namely ***Diplostomum pseudospathaceum*** Niewiadomska, 1984 (Figure 6a), ***Di. spathaceum*** (Rudolphi, 1819) (Figure 6b), ***Posthodiplostomum cuticola*** (von Nordmann, 1832), ***Tylodelphys clavata*** (von Nordmann, 1832) (Figure 6c) and an unidentified species, ***Tylodelphys* sp.** Species delimitation and identification of metacercariae of these species was based solely on the *cox*1 mtDNA sequence analyses. The dataset used for analyses was limited to the species found in the present study and the sequences of the same species retrieved from GenBank (Figure 7). All diplostomid species recorded in the present study are parasites of piscivorous birds.

Metacercariae of *Diplostomum* spp. (Figure 6a,b) were found in the eye lenses of *N. melanostomus* from the Curonian Lagoon [Kiaulės nugara (P = 6 out of 7, 1–76), Klaipėda (P = 84%, 1–64) and Ventė (P = 90%, 8–80)]; in the eye lenses of *N. fluviatilis* from the Kaunas Water Reservoir (P = 93%, 1–11), as well as the Jūra (P = 33%, 1–2), Neris (Buivydžiai, P = 7%, 1) and Nemunas [Sudargas (P = 1 out of 1, 2) and Vilkija (P = 35%, 1–9)] rivers; and in the eye lenses of *Ps. parva* in the Pilvė (P = 82%, 1–5) and Upė (P = 6%, 1) rivers. The hosts for metacercariae of *Di. pseudospathaceum* were *N. fluviatilis* and *N. melanostomus*. *Diplostomum spathaceum* was the only parasite recorded in all three infected fish hosts. Metacercariae of *Di. pseudospathaceum* were previously reported in *N. fluviatilis* in its native and non-native distribution ranges, including the Włocławek Reservoir and the River Vistula (Poland) [51,60]. Metacercariae of *Di. spathaceum* are known to parasitise both gobiid species outside of their native distribution ranges in different waterbodies in Belarus, Poland, Estonia, Germany, Russia, Ukraine, and the USA [51]. In Lithuania, this species was only previously reported in round gobies from the Curonian Lagoon [16].

A single metacercaria of *Po. cuticola* was found on the intestinal wall of *N. fluviatilis* caught in the River Jūra (P = 6%). This is the first report of *Po. cuticola* infecting *N. fluviatilis* in both its native and non-native distribution. Metacercariae of *T. clavata* (Figure 6c) were recorded in the vitreous humour of *N. melanostomus* from the Curonian Lagoon [Kiaulės nugara (P = 5 out of 7, 1–14), Klaipėda (P = 53%, 1–3), and Ventė (P = 50%, 2–34] and *N. fluviatilis* from the Kaunas Water Reservoir (P = 23%, 1–6). Prior to our study, metacercariae of *T. clavata* were known to parasitise both gobiid species outside their native distribution ranges in different waterbodies in Austria, Estonia, Germany, Poland, Slovakia, Russia, and Ukraine [51].

An unidentified species, *Tylodelphys* sp. was reported only once in the vitreous humour of *Ps. parva* collected from a swamp near Dvarviečiai (P = 6%, 1–2).

**Figure 8:**
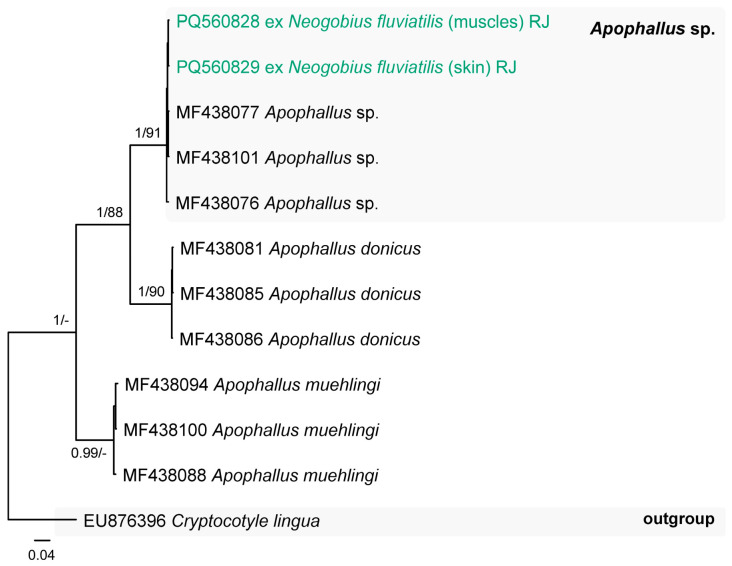
Phylogram resulting from Bayesian inference (BI) analysis based on the *cox*1 mtDNA sequences of the members of *Apophallus* with nodal support values shown at the node as BI/ML (maximum likelihood). Only values > 0.90 (BI) and >70 (ML) are displayed. Scale bar indicates the expected number of substitutions per site. Sequences generated in this study are in the colour corresponding to the colour of their fish host (see Figure 1, Table 1 for abbreviations).

A single member of the family Heterophyidae, the unidentified species ***Apophallus* sp.**, was found in *N. fluviatilis* from the Kaunas Water Reservoir (P = 3%, 3), as well as from the Jūra (P = 11%, 1–48) and Nemunas (Vilkija, P = 3%, 1) rivers. This species was identified based on the *cox*1 mtDNA sequence analyses (Figure 8). The representatives of the genus *Apophallus* parasitise birds and mammals [61].

**Figure 9:**
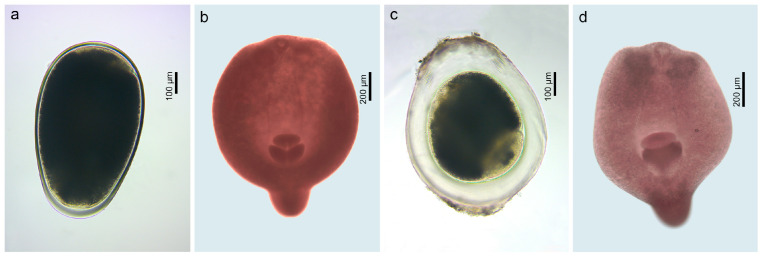
Microphotographs of the helminth parasites found in invasive fish in the present study: (**a**) *Apatemon* sp. 7, live, encysted ex *N. fluviatilis* (voucher, PQ560832); (**b**) *Apatemon* sp. 7, fixed, excysted ex *N. fluviatilis* (voucher, PQ560830); (**c**) *Apatemon* sp. 8, live, encysted ex *N. melanostomus* (voucher, PQ560838); (**d**) *Apatemon* sp. 8, fixed, excysted ex *N. fluviatilis* (voucher, PQ582087, PQ560836).

Metacercariae of two species of the genus *Apatemon* (Strigeidae) were found in both species of *Neogobius*: in *N. melanostomus* from the Curonian Lagoon (Ventė, P = 20%, 1–7); and in *N. fluviatilis* from the Kaunas Water Reservoir (Grabuciškės, P = 7%, 1) and from the Jūra (P = 11%, 1–5), Nemunas [Sudargas (P = 1 out of 1, 1) and Vilkija (P = 29%, 1–12)] and Neris [Buivydžiai (P = 33%, 1–8) and Skirgiškės (P = 19%, 1–3)] rivers. Metacercariae of ***Apatemon* sp. 7** (Figure 9a,b) infected a variety of host tissues (gills, brain, heart, and intestinal wall) in *N. fluviatilis*, while metacercariae of ***Apatemon* sp. 8** (Figure 9c,d) only infected the eyes of *N. fluviatilis* and *N. melanostomus*. 

**Figure 10:**
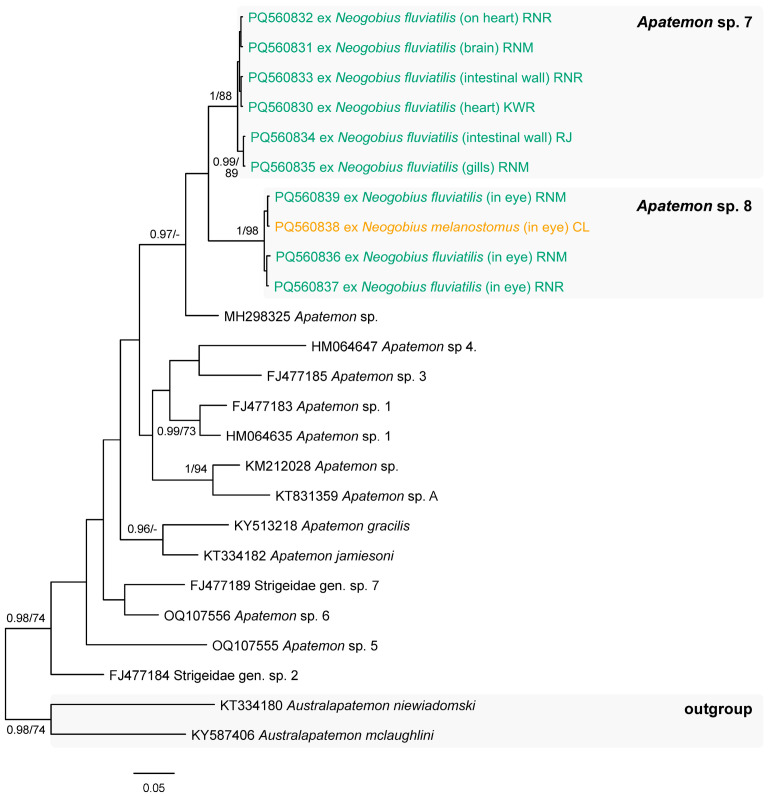
Phylogram resulting from Bayesian inference (BI) analysis based on the *cox*1 mtDNA sequences of the members of *Apatemon* with nodal support values shown at the node as BI/ML (maximum likelihood). Only values > 0.90 (BI) and >70 (ML) are displayed. Scale bar indicates the expected number of substitutions per site. Sequences generated in this study are in the colour corresponding to the colour of their fish host (see Figure 1, Table 1 for abbreviations).

Species delimitation and identification of *Apatemon* were based on the *cox*1 mtDNA sequence analyses (Figure 10). Prior to our study, metacercariae of *Apatemon gracilis* (Rudolphi, 1819) were reported from the monkey and round gobies within their native distribution, as well as outside their native ranges from different freshwater bodies in Bulgaria, Germany, Hungary, Slovakia, Poland, Russia, and Ukraine [51]. Isolates of both species of the present study did not show close affinities with the isolate of *Ap. gracilis* (Figure 10). This suggests that at least three species of the genus *Apatemon* parasitise round and monkey gobies outside their native distribution ranges. The two species of *Apatemon* found here represent new species reports in the studied hosts. The species of the genus *Apatemon* are parasites of piscivorous birds.

**Class Monopisthocotyla (former Class Monogenea)**

**Figure 11:**
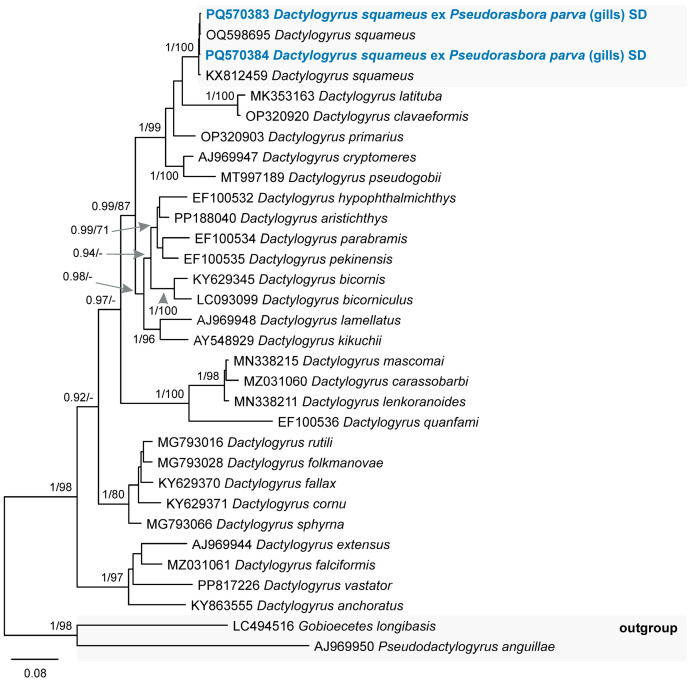
Phylogram resulting from Bayesian inference (BI) analysis based on the 28S rDNA sequences of the members of *Dactylogyrus* with nodal support values shown at the node as BI/ML (maximum likelihood). Only values > 0.90 (BI) and >70 (ML) are displayed. Scale bar indicates the expected number of substitutions per site. Sequences generated in this study are in bold and the colour corresponds to the colour of their fish host (see Figure 1, Table 1 for abbreviations).

Two species of monogeneans were found in the present study. ***Dactylogyrus squameus*** Gusev, 1955 (Dactylogyridae) was found in the gills of *Ps. parva* from a swamp near Dvarviečiai (P = 12%, 2–4) and ***Gyrodactylus proterorhini*** Ergens, 1967 (Gyrodactylidae) was found in the gills of *N. fluviatilis* from the Kaunas Water Reservoir (P = 3%, 2) and the River Neris [Buivydžiai (P = 13%, 5–49) and Skirgiškės (P = 19%, 1–7)]. Species identification of *Da. squameus* was based on the 28S rDNA sequence analyses (Figure 11) whereas the identification of *G. proterorhini* was based on the ITS2 sequences comparison. Our sequences of *G. proterorhini* differed from sequences of *G. proterorhini* from the tubenose goby *Proterorhinus semilunaris* (Heckel, 1937) in Bulgaria (GenBank number MK584285) and in Belgium (GenBank number KR869884) by 0.8% (4 nucleotides), which is considered an intraspecific variation.

The native distribution of *Da. squameus* is within China; however, it is known to have been accidentally introduced to waterbodies in Czechia, Italy and Ukraine [62]. Our study reports on *Da. squameus* in *Ps. parva* for the first time in Lithuania. The native distribution of *G. proterorhini* is the Black and Azov seas and their estuaries [63]; however, it has been reported from the monkey goby outside of its native range of distribution in freshwater ecosystems in Bulgaria, Germany, Poland, and Ukraine [51].

**Phylum Nematoda**
**Class Adenophorea**
**Class Secernentea**

The classification of nematodes presented here is that of Moravec [64].

**Figure 12:**
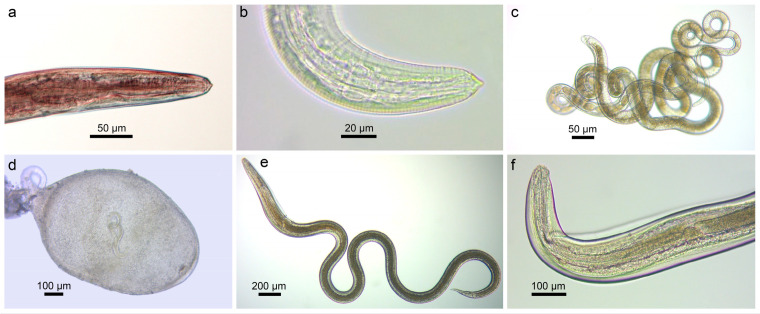
Microphotographs of nematodes found in invasive fish in the present study: (**a**) anterior end of *Streptocara crassicauda*, fixed ex *N. fluviatilis*; (**b**) anterior end of *Syncuaria* sp., live ex *N. melanostomus*; (**c**) *Pseudocapillaria tomentosa*, live ex *Ps. parva*; (**d**) encapsulated second-stage larva of *Raphidascaris acus* in liver, live ex *N. fluviatilis* (voucher, PQ570395); (**e**) whole worm and (**f**) anterior end of third-stage larva of *Raphidascaris acus* on liver, exsheathed, live ex *N. fluviatilis* (voucher, PQ570398).

A total of six species of nematodes belonging to five families, namely the Acuariidae, Raphidascarididae, and Rhabdochonidae (Secernentea) and Capillariidae, and Dioctophymatidae (Adenophorea), were found in the present study (Figure 12). 

***Streptocara crassicauda*** (Creplin, 1829) (Acuariidae) (Figure 12a) was found in *N. fluviatilis* from the River Nemunas (Vilkija, P = 3%, 1). This species of nematode is common in both gobiid species in their native distribution ranges and has been reported in the round goby from the Danube (Slovakia and Austria) and Rhine (Germany) rivers [51]. ***Syncuaria* sp.** (Figure 12b) (Acuariidae) was found in *N. melanostomus* from the Curonian Lagoon (Ventė, P = 10%, 4). Our study is the first to report species of this genus in the round goby. ***Eustrongylides excisus*** Jägerskiöld, 1909 (Dioctophymatidae) was found in *N. fluviatilis* from the Kaunas Water Reservoir (P = 50%, 1–3), as well as from the Jūra (P = 11%, 1) and Nemunas (Vilkija, P = 3%, 1) rivers. This species of nematode is common in both gobiid species in their native distribution ranges, as well as in their non-native ranges in freshwater bodies in Austria, Czechia, Estonia, Germany, Poland, Russia, and Ukraine [51]. The definitive host of *Eus. excisus*, *St. crassicauda*, *Syncuaria* sp. are birds, while fish serve as paratenic hosts [65].

Another species found in monkey gobies from the Nemunas (Vilkija, P = 3%, 2) and Neris (Buivydžiai, P = 7%, 3) rivers was ***Rhabdochona* sp.** (Rhabdochonidae). Nematodes of ***Raphidascaris acus*** (Bloch, 1779) (Raphidascarididae) (Figure 12d–f) were found in both *N. fluviatilis* from the Jūra (P = 6%, 3), Nemunas (Vilkija, P = 21%, 1–4) and Neris (Skirgiškės, P = 7%, 1) rivers, and *N. melanostomus* from the Curonian Lagoon (Kiaulės nugara, P = 1 out of 7, 1). This species of nematode is common in both gobiid species in their native distribution ranges as well as in their non-native ranges in waterbodies in Austria, Croatia, Czechia, Germany, Hungary, France, Slovakia, Ukraine, and the USA [51]. In Europe, *Ra. acus* uses pike *Esox lucius* Linnaeus, 1758 as its main host; adult worms are also found in fish from the Anguillidae, Lotidae, Percidae, and Salmonidae, and occurrence in other fishes is rare [65]. Larvae of *Ra. acus* were found in various fish species of different families [65]. The only species of nematode found in *Ps. parva* from the River Pilvė (P = 27%, 2–6) and a swamp near Dvarviečiai (P =24%, 1–4) was ***Pse. tomentosa*** (Capillariidae) (Figure 12c). This nematode is distributed widely in Palearctic Eurasia and North America, has a direct life cycle and a wide variety of freshwater fish can serve as its definitive hosts [64].

**Figure 13:**
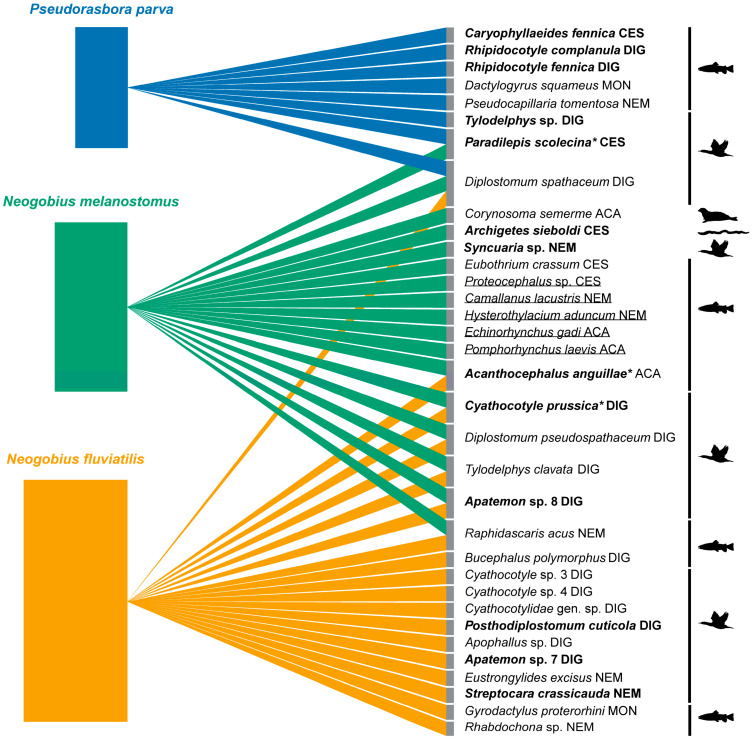
Graphical representation of the invasive fish species and their helminth parasites in Lithuania based on the data obtained in the present study and previous studies in Lithuania [16,22]. Each rectangle represents a host species with its size corresponding to the total number of fish examined during this study. Helminth species in bold represent new host records: * only *Ps. parva* is a new host for *Paradilepis scolecina*, *N. fluviatilis* is a new host for *Acanthocephalus anguillae* and *N. melanostomus* is a new host for *Cyathocotyle prussica*. Silhouettes of a bird, fish, oligochaete, and seal represent the definitive hosts of recorded species. Species found in previous studies conducted in Lithuania are underlined. Abbreviations: (ACA) Acanthocephala; (CES) Cestoda; (DIG) Digenea; (MON) Monogenea; (NEM) Nematoda.

When comparing the helminth diversity of each fish species within all examined waterbodies, we found that *N. fluviatilis* collected from the River Nemunas contained the highest helminth diversity with 13 species, followed by 9 species from the River Neris, 8 species from the River Jūra and 7 species from the Kaunas Water Reservoir. The highest number of helminth species in *Ps. parva* was recorded in a swamp near Dvarviečiai (five species), followed by the Pilvė and Upė rivers, which contained three helminth species each. There was a clear separation of the helminth community of *Ps. parva* from those of the two gobiid species, with a 24% overlap in the parasite communities of *N. fluviatilis* and *N. melanostomus* (Figure 13).

The core helminth community of *N. fluviatilis* consisted of three species of digenean trematodes, *B. polymorphus* (registered at four out of six sites, P = 6–30%, 1–44), *Apatemon* sp. 7 (at five out of six sites, P = 7–20%, 1–7) and *Di. spathaceum* (at five out of six sites). Contrastingly, the key species in the helminth community of *N. melanostomus* were two species of digenean trematodes, *Di. spathaceum* (registered at three out of three sites) and *T. clavata* (registered at three out of three sites, P = 50–53%, 15). The core helminth community of *Ps. parva* consisted of two species of digenean trematodes, *Di. spathaceum* (registered at two out of three sites, P = 6–82%, 1–5), *Rhi. fennica* (registered at two out of three sites, P = 82%, 1–76) and one species of nematode, *Pse. tomentosa* (registered at two out of three sites, P = 24–27%, 1–6). The most common helminths were metacercariae of *Diplostomum* spp., occurring in the eye lenses of all 3 infected fish species in 10 out of the 12 examined sampling sites.

Generally, the infection rates among fish species and waterbodies examined exhibited similar trends. A total of 68% of examined individuals of *Ps. parva* (n = 62) and 79% of examined *N. fluviatilis* (n = 125) were infected. *Neogobius melanostomus* (n = 36) supported the highest prevalence of infection at 89%. The highest helminth prevalence in *N. fluviatilis* (100%) was observed at the sampling site of Grabuciškės in the Kaunas Water Reservoir, in *N. melanostomus* (90%) at the sampling site of Ventė in the Curonian Lagoon, and in *Ps. parva* (91%) at the sampling sites of Antanavas in the River Pilvė and a swamp near Dvarviečiai.

## 4. Discussion

Characterising the helminth communities of invasive fish is important for understanding their role in the waterbodies within which they have been introduced, as they can introduce new parasites that may be transmitted to native hosts (spillover), leading to the emergence of new diseases and potential socio-economic impacts. Alternatively, if invasive fish become suitable hosts for local parasites, they may contribute to parasite dynamics by either increasing the infections of native fish through transmission amplification (spillback) or by decreasing the infections of native fish through transmission reduction (dilution).

The present study reports on 29 helminth species parasitising three invasive fish species, *N. fluviatilis*, *N. melanostomus,* and *Ps. parva,* occurring in Lithuanian freshwater ecosystems. Since their introduction, these fish have become competent hosts for 22 local fish helminths [66]; our unpublished data], confirming that spillback processes are taking place. Out of the seven remaining helminth species identified in this study, two (monogeneans, *Da. squameus,* and *G. proterorhini*) were co-introduced with their fish hosts, while five (digeneans, *Apatemon* sp. 7, *Apatemon* sp. 8, *Cyathocotyle* sp. 4, cestode *Ar. sieboldi*, and nematode *Syncuaria* sp.), which have not been documented in these fishes’ native distribution ranges, were acquired in the invasive range and were exclusively found in these invasive fish species.

Out of the 18 helminth species found in *N. fluviatilis*, 14 (78%) were native fish helminths. Of these, six species (43%)—*B. polymorphus*, *Di. spathaceum*, *T. clavata*, *St. crassicauda*, *Eus. excisus*, and *Ra. acus*—were known to parasitise *N. fluviatilis* in its native distribution range [51]. These helminths are common species that utilise a wide spectrum of piscivorous birds or predatory fish as definitive hosts. A monogenean species *G. proterorhini* was co-introduced with its fish host from its native distribution. The three remaining species recorded in this study—*Apatemon* sp. 7, *Apatemon* sp. 8, and *Cyathocotyle* sp. 4—have never been reported in *N. fluviatilis*, neither in their native nor invasive distribution ranges. As adults, these helminths parasitise birds and, although they have not been reported in native fish, they seem to successfully use the invasive monkey goby as a competent host for their development and transmission to definitive hosts. This finding brings the total number of helminths reported from *N. fluviatilis* in its invasive range up to 53, which is lower than the helminth richness reported from its native range (77 species) [51].

The helminth diversity of *N. melanostomus* consisted of 11 species, of which 8 (73%) represent native fish helminths. Of these, five species (63%)—*Pa. scolecina*, *Di. pseudospathaceum*, *Di. spathaceum*, *T. clavata*, and *Ra. acus*—are known parasites of *N. melanostomus* in their native distribution range. Two species—*Ar. sieboldi* and *Syncuaria* sp.—were found exclusively in *N. melanostomus* and one species, *Apatemon* sp. 8, was shared with *N. fluviatilis*. To date, the cestode *Ar. sieboldi* has not been reported in native fish (intermediate hosts) or oligochaetes (definitive hosts) in Lithuania, and this represents the first record of this species in the invasive monkey goby. At least 26 species of oligochaetes occur in the Lithuanian part of the Curonian Lagoon [67], including naidid oligochaetes that are known as definitive hosts for *Ar. sieboldi*. Another species that has not previously been recorded in native fish and the round goby is the nematode *Syncuaria* sp. Nematodes of the genus *Syncuaria* are parasites of aquatic birds, including phalacrocoracids, with freshwater fish serving as their paratenic hosts. Considering several large colonies of great cormorants residing at the Curonian Lagoon and a previous record of *Syncuaria squamata* (Linstow, 1883) infecting these birds [56], they could potentially act as the definitive hosts of *Syncuaria* sp. recorded in the round goby in the present study. New records of these three species increase the diversity of helminths in the round goby in its invasive range up to 97, which is lower compared to that reported in its native range (115 species) [51]. 

Prior to our study, seven helminth species were reported in *N. melanostomus* from the Curonian Lagoon. Since then (16 years), this number has increased to 17. A single species, *Di. spathaceum*, was found in a previous study [16] as well as in the present study. Hence, the total number of helminths infecting both invasive gobiids, *N. melanostomus* and *N. fluviatilis,* in Lithuania is similar (17 and 18 species, respectively). However, the helminth fauna of *N. melanostomus* is much more taxonomically diverse, consisting of digeneans (5), acanthocephalans (4 species), cestodes (4) and nematodes (4), while the faunal diversity of *N. fluviatilis* is dominated by digeneans (12), with fewer species of nematodes (4), acanthocephalans (1) and monogeneans (1). Despite both species having similar feeding habits and occurring in similar habitats, the differences in their helminth fauna could be related to the diversity of hosts (definitive and intermediate) sharing the ecosystems that these fish species inhabit. The diversity and abundance of fish and birds, including migratory birds, which serve as definitive hosts for the majority of species recorded in the present study (Figure 13), are higher in the Curonian Lagoon compared to the riverine habitats from where *N. fluviatilis* was sampled. Seven taxa, with digenean being the most diverse group (digenean trematodes —5 species, an acanthocephalan—1, and a nematode—1) (Figure 13), were shared between the gobiid fishes, representing almost half of their helminth fauna (39% for *N. fluviatilis* and 41% for *N. melanostomus*). The highest diversity of helminth species recorded in *N. fluviatilis* was from the Nemunas River basin (13 species); however, it differed amongst sampling sites. The highest diversity was at the Vilkija site of the Nemunas River (11 species), followed by the Grabuciškės site of the Kaunas water reservoir (7 species), and the Mociškiai site of the River Jūra (7 species). The highest diversity of helminth species in *N. melanostomus* was detected at the Klaipėda site of the Curonian Lagoon (7 species), the locality where this fish species first entered the lagoon [67].

Based on the examination of the stone mokoro, *Ps. parva*, our study suggests that this fish seemed to be introduced to Lithuanian freshwaters without any endohelminths. Subsequently, it has acquired seven native fish helminths (spillback). The helminth fauna of *Ps. parva* consisted of digenean trematodes (5), a cestode (1), a monogenean (1), and a nematode (1) (Figure 13). There was a clear separation of the helminth fauna of *Ps. parva* from those of the two gobiid species, with only two species (*Di. spathaceum* and *Pa. scolecina*) overlapping (Figure 13). However, *Ps. parva* was co-introduced with a monogenean species, *Da. squameus*, from its native distribution range in China. Although none of the native fish (more than 900 individuals of 38 species examined; our unpublished data) were found to be infected with *Da. squameus*, this finding is worrisome since possible host switches to native fish may have serious consequences [8]. Monogeneans of *Da. squameus* were recently reported in *Ps. parva* in its invasive distribution range in Europe from Czechia, Italy, and Ukraine [62]. Similarly to all previous reports, the prevalence and intensity of infection with this species in the present study was low (P = 12%, 2–4 specimens per fish). Furthermore, apart from *Da. squameus*, Ondračková et al. [62] reported two additional species of monogeneans parasitising *Ps. parva* in its invasive distribution range in Europe that were not detected in the present study.

Another study reporting on the parasite fauna of *Ps. parva* was conducted in neighbouring Poland [68,69], where *Ps. parva* was infected with 13 helminth species: 5 digenean trematodes (*Ap. gracilis*, *Phyllodistomum elongatum* Nybelin, 1926, *Phy. folium* (Olfers, 1816), *Po. cuticola* and an unidentified metacercaria), four nematodes (*Cystidicola farionis* Fischer, 1798, *Pse. tomentosa*, *Rhabdochona ergensi* Moravec, 1968, and Anisakidae gen. sp. larvae), three monogeneans [*Bivaginogyrus obscurus* (Gusev, 1955), *Da. squameus* and *Diplozoon paradoxum* von Nordmann, 1832], and a cestode [*Caryophyllaeus laticeps* (Pallas, 1781)]. Out of these, only two species were found in *Ps. parva* from Lithuania. Compared to the previous studies reporting both gobiid species and the stone mokoro as hosts for adult endohelminths in their invasive distribution range [51,69,70], these fish were found to serve as intermediate or paratenic hosts (except nematode *Pse. tomentosa*) in the present study.

All examined individuals of *Pe. glenii* in our study were uninfected with helminth parasites. However, two species of digenean metacercariae (*Metorchis xanthosomus* (Creplin, 1846) and an unidentified Strigeidae gen. sp.), with low infection rates, were recently reported from neighbouring Latvia [71], while thirteen helminth species (1 cestode, 7 digenean, 3 nematodes, and 2 monogeneans) were reported from *Pe. glenii* in Poland [72]. Interestingly, one of the monogeneans, *G. proterorhini*, reported in *Pe. glenii* in Poland was found in our study parasitising the monkey goby. *Gyrodactylus proterorhini* was originally described from the tubenose goby *Pr. semilunaris* and has since then been reported from other gobiids occurring in the Black and Azov seas and their estuaries [73]. The report of this parasite from *Pe. glenii*, demonstrating that it can easily switch hosts, might therefore be a potential threat to native fish in Lithuania.

Amongst the helminth species reported in our study, *Eus. excisus* is a fish-borne zoonotic nematode that infects numerous freshwater fish species and fish-eating birds from the families Phalacrocoracidae, Ardeidae, Ciconiidae, Pelicanidae, etc. Prior to our study, this species had neither been reported in native nor invasive fish in Lithuania. We detected this species in several native fish including, *Abramis brama* (Linnaeus, 1758) (2 instances), *Perca fluviatilis* Linnaeus, 1758 (7 instances), *Platichthys flesus* (Linnaeus, 1758) (2 instances), *Pungitius pungitius* (Linnaeus, 1758) (2 instances) and *Silurus glanis* (Linnaeus, 1758) (3 instances) (our unpublished data). The infection of *N. fluviatilis* with *Eus. excisus* was recorded more often (18 instances, P = 14%), demonstrating its key role in transmitting this nematode species in freshwater ecosystems in Lithuania.

By assessing the current helminth diversity in invasive fish in Lithuania, this study is the first step toward further investigations of the potential risks these species represent to native ecosystems. The fact that we reported new helminth species in *Neogobius* spp. and *Ps. parva* in their invasive distribution ranges suggests that these species are integrating well into their newly invaded ecosystems and can be considered as distribution agents of common native helminth parasites.

## 5. Conclusions

Understanding the roles of invasive fish species on local ecosystems is crucial in a world where invasion is accelerating at an ever-increasing rate. By assessing the helminth diversity of invasive freshwater fish in Lithuania, a region where such studies are virtually lacking, our study revealed relatively diverse helminth fauna consisting of 29 helminth taxa. Out of the four examined fish species, *N. fluviatilis* appeared to support the highest helminth diversity followed by *N. melanostomus* and *Ps. parva*, whereas *Pe. glenii* was free from infection with helminths. This study provides comprehensive data on helminth parasites of invasive fish from Lithuania, which will further be used to evaluate the impact of invasive fish on native fish species and their parasite communities. Additionally, the present study demonstrated the importance of molecular identification of helminths in freshwater fish as they are often present at their larval stages that lack reliable morphological features for species identification.

## Figures and Tables

**Table 1:** Summary data of sampling sites and fish species examined.

Fish Species	Water Body/Locality	Coordinates	Sample Size	TL ± SD	Q ± SD	Age, Min–Max	No. of Infected Fish (P)
*Neogobius fluviatilis*	**Kaunas Water Reservoir** (KWR)						
	Grabuciškės	54.89238, 24.146082	30	11.1 ± 1.5	16.1 ± 8.3	3–5	30 (100)
	**River Jūra** (RJ)						
	Mociškiai	55.107601, 22.173838	18	9.1 ± 1.3	6.3 ± 3.0	2–4	11 (61)
	**River Nemunas** (RNM)						
	Vilkija	55.029198, 23.58906	34	10.0 ± 1.4	9.7 ± 5.0	2–5	25 (74)
	Sudargas	55.051171, 22.643179	1	10.7	10.0	3	1
	**River Neris** (RNR)						
	Buivydžiai	54.863282, 25.745817	15	9.8 ± 1.5	9.7 ± 3.8	1–3	10 (67)
	Skirgiškės	54.837858, 25.375848	27	8.7 ± 1.3	6.0 ± 3.2	1–4	22 (81)
*Neogobius melanostomus*	**Curonian Lagoon** (CL)						
	Kiaulės nugara	55.656278, 21.135288	7	14.5 ± 3.0	52.9 ± 32.2	4–8	6
	Klaipėda	55.718409, 21.102401	19	15.0 ± 2.7	55.3 ± 30.5	4–9	17 (89)
	Ventė	55.346829, 21.194767	10	9.1 ± 2.1	12.1 ± 9.3	2–5	9 (90)
*Perccottus glenii*	**Lake Bedugnis**						
	Vievis	54.783468, 24.812681	10	13.1 ± 2.6	29.8 ± 15.5	3–7	0
	**Lake Gabrio**						
	Laukesa	55.763536, 26.276487	6	17.2 ± 6.4	88.5 ± 75.9	2–10	0
	**Lake Šėlinis**						
	Kochanovka	55.173501, 26.258422	18	10.3 ± 2.0	13.6 ± 7.6	2–5	0
	Pond in Alytus	54.400521, 24.009722	19	12.7 ± 2.1	24.6 ± 13.8	2–6	0
	**River Merkys**						
	Pamerkiai	54.425358, 25.552918	1	9.6	12.0	3	0
	**River Neris** (RNR)						
	Skirgiškės	54.837858, 25.375848	1	6.3	3.3	1	0
*Pseudorasbora parva*	**River Upė** (RU)						
	Paupys	55.320702, 22.894767	18	6.2 ± 0.6	1.9 ± 0.6	1–3	2 (11)
	**River Pilve** (RP)						
	Antanavas	54.713178, 23.319018	11	8.4 ± 0.9	5.0 ± 1.2	2–5	10 (91)
	Swamp Dvarviečiai (SD)	56.110158, 21.824709	33	8.1 ± 0.6	4.7 ± 1.3	2–5	30 (91)

(TL) total length (cm); (SD) standard deviation; (Q) total weight (g); (P) prevalence of infection (%).

**Table 2:** Primers and protocols used for PCR amplification and sequencing in this study.

Gene	Name		Sequence	FSL	Taxa	Reference
18S rRNA	18SU467F	F	5′-ATCCAAGGAAGGCAGCAGGC-3′	815	A	[29]
	18SL1310R	R	5′-CTCCACCAACTAAGAACGGC-3′			[29]
28S rRNA	LSU5	F	5′-TAGGTCGACCCGCTGAAYTTAAGCA-3′	999	A	[30]
	1200R	R	5′-GCATAGTTCACCATCTTTCGGG-3′			[31]
	ZX-1	F	5′-ACCCGCTGAATTTAAGCATAT-3′	1220–1282	C, D	[32]
	1500R	R	5′-GCTATCCTGAGGGAAACTTCG-3′			[33]
	C1	F	5′-ACCCGCTGAATTTAAGCA-3′	818–845	M	[34]
	D2	R	5′-TGGTCCGTGTTTCAAGAC-3′			[34]
	U178	F	5′-GCACCCGCTGAAYTTAAG-3′	1244–1498	M	[31]
	L1642	R	5′-CCAGCGCCATCCATTTTCA-3′			[31]
	CTEf	F	5′-AGTGAATGGGGAAAAGCCCA-3′	921	N	[35]
	CTEr	R	5′-GACCTCCACCAGAGTTTCC-3′			[35]
ITS2	D	F	5′-GGCTYRYGGNGTCGATGAAGAACGCAG-3′	595	M	[36]
	B1	R	5′-GCCGATCCGAATCCTGGTTAGTTTCTTTTCCT-3′			[36]
*cox*1	Dice1F	F	5′-ATTAACCCTCACTAAATTWCNTTRGATCAT AAG-3′	756–820	D	[37]
	Dice14R	R	5′-TAATACGACTCACTATACCHACMRTAAACATAT GATG-3′			[37]
	Dig_cox1Fa	F	5′-ATGATWTTYTTYTTYYTDATGCC-3′	461–515	D	[38]
	Dig_cox1R	R	5′-TCNGGRTGHCCRAARAAYCAA AA-3′			[38]

(F) forward; (R) reverse; (FSL) final sequences length (nt); (A) Acanthocephala; (C) Cestoda; (D) Digenea; (M) Monogenea; (N) Nematoda.

## Data Availability

Data are available upon request from the corresponding authors.

## Supplementary Materials

The following supporting information can be downloaded at: https://www.mdpi.com/article/10.3390/ani14223293/s1, Table S1: summary data for the sequences of helminths generated in the present study.
