# Supplementary material for: Helminth Parasites of Invasive Freshwater Fish in Lithuania
Source: Animals (Basel). 2024 Nov 15;14(22):3293. doi: 10.3390/ani14223293 (PMC11591254; doi:10.3390/ani14223293)
Supplement: Supplementary file 1 [file animals-14-03293-s001.zip › animals-3300915-supplementary.pdf]

Helminth parasites of invasive freshwater fish in Lithuania

Olena Kudlai, Vytautas Rakauskas, Nathan Jay Baker, Camila Pantoja, Olga Lisitsyna, Rasa Binkienė

Table S1. Summary data for the sequences of helminths generated in the present study

Species	Stage	Isolate	Host	Locality	GenBank accession numbers	
					28S	*cox*1
Acanthocephala						
Family Polymorphidae						
Corynosoma semerme	cystacanth	FP393	*Neogobius melanostomus*	Curonian Lagoon, Klaipėda	PQ570008 (PQ557260)*	–
Cestoda						
Family Caryophyllaeidae						
Archigetes sieboldi	larva	FP396	*Neogobius melanostomus*	Curonian Lagoon, Klaipėda	PQ570009	–
Family Gryporhynchidae						
Paradilepis scolecina	larva	FP395	*Neogobius melanostomus*	Curonian Lagoon, Klaipėda	PQ570010	–
Family Lytocestidae						
Caryophyllaeides fennica	larva	FP344	*Pseudorasbora parva*	River Upė, Paupys	PQ570011	–
Family Triaenophoridae						
Eubothrium crassum	juvenile adult	FP390	*Neogobius melanostomus*	Curonian Lagoon, Klaipėda	PQ570012	–
Digenea						
Family Bucephalidae						
Bucephalus polymorphus	metacercaria	FP177	*Neogobius fluviatilis*	Kaunas water reservoir, Grabuciškės	PQ582077	PQ560762
	metacercaria	FP178	*Neogobius fluviatilis*	Kaunas water reservoir, Grabuciškės	–	PQ560763
	metacercaria	FP183	*Neogobius fluviatilis*	River Neris, Skirgiškės	–	PQ560764
	metacercaria	FP214	*Neogobius fluviatilis*	Kaunas water reservoir, Grabuciškės	–	PQ560765
	metacercaria	FP403	*Neogobius fluviatilis*	River Neris, Skirgiškės	–	PQ560766
	metacercaria	FP404	*Neogobius fluviatilis*	River Neris, Skirgiškės	PQ582078	PQ560767
	metacercaria	FP448	*Neogobius fluviatilis*	River Neris, Skirgiškės	–	PQ560768
Rhipidocotyle campanula	metacercaria	FP549	*Pseudorasbora parva*	Swamp near Dvarviečiai	PQ582079	PQ560769
	metacercaria	FP605	*Pseudorasbora parva*	Swamp near Dvarviečiai		PQ560770
Rhipidocotyle fennica	metacercaria	FP547	*Pseudorasbora parva*	Swamp near Dvarviečiai	–	PQ560771
	metacercaria	FP548	*Pseudorasbora parva*	Swamp near Dvarviečiai	–	PQ560772
	metacercaria	FP551	*Pseudorasbora parva*	Swamp near Dvarviečiai	–	PQ560773
	metacercaria	FP552	*Pseudorasbora parva*	Swamp near Dvarviečiai	PQ582080	PQ560774
	metacercaria	FP553	*Pseudorasbora parva*	Swamp near Dvarviečiai	–	PQ560775
	metacercaria	FP554	*Pseudorasbora parva*	Swamp near Dvarviečiai	–	PQ560776
	metacercaria	FP603	*Pseudorasbora parva*	Swamp near Dvarviečiai	–	PQ560777
	metacercaria	FP604	*Pseudorasbora parva*	Swamp near Dvarviečiai	–	PQ560778
	metacercaria	FP756	*Pseudorasbora parva*	River Pilvė, Antanavas	–	PQ560779
	metacercaria	FP759	*Pseudorasbora parva*	River Pilvė, Antanavas	–	PQ560780
	metacercaria	FP908	*Pseudorasbora parva*	River Pilvė, Antanavas	–	PQ560781

Table S1 continued

Species		Isolate	Host	Locality	GenBank accession numbers	
					28S	*cox*1
Family Cyathocotylidae						
Cyathocotyle prussica	metacercaria	FP175	*Neogobius fluviatilis*	River Nemunas, Vilkija	–	PQ560782
	metacercaria	FP212	*Neogobius fluviatilis*	River Nemunas, Vilkija	PQ582081	–
	metacercaria	FP215	*Neogobius fluviatilis*	River Nemunas, Vilkija	–	PQ560783
	metacercaria	FP405	*Neogobius fluviatilis*	River Neris, Skirgiškės	–	PQ560784
	metacercaria	FP407	*Neogobius fluviatilis*	River Neris, Skirgiškės	–	PQ560785
	metacercaria	FP409	*Neogobius fluviatilis*	River Neris, Skirgiškės	–	PQ560786
	metacercaria	FP411	*Neogobius fluviatilis*	River Neris, Skirgiškės	–	PQ560787
	metacercaria	FP536	*Neogobius fluviatilis*	River Nemunas, Vilkija	–	PQ560788
	metacercaria	FP537	*Neogobius fluviatilis*	River Nemunas, Vilkija	–	PQ560789
	metacercaria	FP910	*Neogobius fluviatilis*	River Nemunas, Vilkija	–	PQ560790
	metacercaria	FP379	*Neogobius melanostomus*	Curonian Lagoon, Ventė	–	PQ560791
	metacercaria	FP383	*Neogobius melanostomus*	Curonian Lagoon, Ventė	–	PQ560792
	metacercaria	FP389	*Neogobius melanostomus*	Curonian Lagoon, Ventė	–	PQ560793
	metacercaria	FP398	*Neogobius melanostomus*	Curonian Lagoon, Klaipėda	–	PQ560794
Cyathocotyle sp. 3	metacercaria	FP546	*Neogobius fluviatilis*	River Nemunas, Vilkija	PQ582082	PQ560795
Cyathocotyle sp. 4	metacercaria	FP174	*Neogobius fluviatilis*	River Nemunas, Vilkija	PQ582083	PQ560796
Cyathocotylidae gen. sp.	metacercaria	FP180	*Neogobius fluviatilis*	Kaunas water reservoir, Grabuciškės	PQ582084	–
Family Diplostomidae						
Diplostomum pseudospathaceum	metacercaria	FP71	*Neogobius fluviatilis*	River Nemunas, Vilkija	–	PQ560797
	metacercaria	FP195	*Neogobius fluviatilis*	River Nemunas, Vilkija	–	PQ560798
	metacercaria	FP475	*Neogobius fluviatilis*	River Jūra, Mociškiai	–	PQ560799
	metacercaria	FP544	*Neogobius fluviatilis*	River Nemunas, Vilkija	–	PQ560800
	metacercaria	FP380	*Neogobius melanostomus*	Curonian Lagoon, Ventė	–	PQ560801
	metacercaria	FP386	*Neogobius melanostomus*	Curonian Lagoon, Ventė	–	PQ560802
	metacercaria	FP387	*Neogobius melanostomus*	Curonian Lagoon, Ventė	–	PQ560803
Diplostomum spathaceum	metacercaria	FP20	*Neogobius fluviatilis*	Kaunas water reservoir, Grabuciškės	–	PQ560804
	metacercaria	FP22	*Neogobius fluviatilis*	River Nemunas, Sudargas	–	PQ560805
	metacercaria	FP72	*Neogobius fluviatilis*	River Nemunas, Vilkija	–	PQ560806
	metacercaria	FP74	*Neogobius fluviatilis*	Kaunas water reservoir, Grabuciškės	–	PQ560807
	metacercaria	FP91	*Neogobius fluviatilis*	River Jūra, Mociškiai	–	PQ560808
	metacercaria	FP196	*Neogobius fluviatilis*	Kaunas water reservoir, Grabuciškės	–	PQ560809
	metacercaria	FP202	*Neogobius fluviatilis*	River Jūra, Mociškiai	–	PQ560810
	metacercaria	FP643	*Neogobius fluviatilis*	River Nemunas, Vilkija	–	PQ560811
	metacercaria	FP75	*Neogobius melanostomus*	Curonian Lagoon, Kiaulės nugara	–	PQ560812
	metacercaria	FP76	*Neogobius melanostomus*	Curonian Lagoon, Kiaulės nugara	–	PQ560813
	metacercaria	FP382	*Neogobius melanostomus*	Curonian Lagoon, Ventė	–	PQ560814
	metacercaria	FP391	*Neogobius melanostomus*	Curonian Lagoon, Klaipėda	–	PQ560815

Table S1 continued

Species		Isolate	Host	Locality	GenBank accession numbers	
					28S	*cox*1
	metacercaria	FP394	*Neogobius melanostomus*	Curonian Lagoon, Klaipėda	–	PQ560816
	metacercaria	FP397	*Neogobius melanostomus*	Curonian Lagoon, Klaipėda	–	PQ560817
	metacercaria	FP758	*Pseudorasbora parva*	River Pilvė, Antanavas	–	PQ560818
Posthodiplostomum cuticola	metacercaria	FP520	*Neogobius fluviatilis*	River Jūra, Mociškiai	–	PQ560819
Tylodelphys clavata	metacercaria	FP98	*Neogobius fluviatilis*	Kaunas water reservoir, Grabuciškės	–	PQ560820
	metacercaria	FP99	*Neogobius fluviatilis*	Kaunas water reservoir, Grabuciškės	–	PQ560821
	metacercaria	FP100	*Neogobius melanostomus*	Curonian Lagoon, Kiaulės nugara	–	PQ560822
	metacercaria	FP101	*Neogobius melanostomus*	Curonian Lagoon, Kiaulės nugara	–	PQ560823
	metacercaria	FP381	*Neogobius melanostomus*	Curonian Lagoon, Ventė	–	PQ560824
	metacercaria	FP384	*Neogobius melanostomus*	Curonian Lagoon, Ventė	–	PQ560825
	metacercaria	FP392	*Neogobius melanostomus*	Curonian Lagoon, Klaipėda	–	PQ560826
Tylodelphys sp.	metacercaria	FP610	*Pseudorasbora parva*	Swamp near Dvarviečiai	–	PQ560827
Family Heterophyidae						
Apophallus sp.	metacercaria	FP474	*Neogobius fluviatilis*	River Jūra, Mociškiai	–	PQ560828
	metacercaria	FP645	*Neogobius fluviatilis*	River Jūra, Mociškiai	PQ582085	PQ560829
Family Strigeidae						
Apatemon sp. 7	metacercaria	FP160	*Neogobius fluviatilis*	Kaunas water reservoir, Grabuciškės	PQ582086	PQ560830
	metacercaria	FP164	*Neogobius fluviatilis*	River Nemunas, Vilkija	–	PQ560831
	metacercaria	FP402	*Neogobius fluviatilis*	River Neris, Skirgiškės	–	PQ560832
	metacercaria	FP406	*Neogobius fluviatilis*	River Neris, Skirgiškės	–	PQ560833
	metacercaria	FP907	*Neogobius fluviatilis*	River Jūra, Mociškiai	–	PQ560834
	metacercaria	FP909	*Neogobius fluviatilis*	River Nemunas, Vilkija	–	PQ560835
Apatemon sp. 8	metacercaria	FP185	*Neogobius fluviatilis*	River Nemunas, Vilkija	PQ582087	PQ560836
	metacercaria	FP408	*Neogobius fluviatilis*	River Neris, Skirgiškės	–	PQ560837
	metacercaria	FP385	*Neogobius melanostomus*	Curonian Lagoon, Ventė	–	PQ560838
	metacercaria	FP545	*Neogobius melanostomus*	River Nemunas, Vilkija	–	PQ560839
Monogenea						
Family Dactylogyridae						
Dactylogyrus squameus	adult	FP606	*Pseudorasbora parva*	Swamp near Dvarviečiai	PQ570383	–
	adult	FP567	*Pseudorasbora parva*	Swamp near Dvarviečiai	PQ570384	–
Family Gyrodactylidae						
Gyrodactylus proterorhini	adult	FP399	*Neogobius fluviatilis*	River Neris, Buivydžiai	PQ570385 (PQ557266)**	–
	adult	FP401	*Neogobius fluviatilis*	River Neris, Skirgiškės	PQ570386	–
	adult	FP441	*Neogobius fluviatilis*	River Neris, Skirgiškės	PQ570387	–
	adult	FP442	*Neogobius fluviatilis*	River Neris, Skirgiškės	PQ570388 (PQ557267)**	–
	adult	FP443	*Neogobius fluviatilis*	River Neris, Skirgiškės	PQ570389	–
	adult	FP640	*Neogobius fluviatilis*	River Neris, Skirgiškės	PQ570390	–

Table S1 continued

Species		Isolate	Host	Locality	GenBank accession numbers	
					**28S**	***cox*1**
	adult	FP641	*Neogobius fluviatilis*	River Neris, Skirgiškės	PQ570391	−
Nematoda						
Family Raphidascarididae						
Raphidascaris acus	larva	FP353	*Neogobius fluviatilis*	River Neris, Skirgiškės	PQ570392	−
	larva	FP355	*Neogobius fluviatilis*	River Nemunas, Vilkija	PQ570393	−
	larva	FP583	*Neogobius fluviatilis*	River Jūra, Mociškiai	PQ570394	−
	larva	FP590	*Neogobius fluviatilis*	River Nemunas, Vilkija	PQ570395	−
	larva	FP591	*Neogobius fluviatilis*	River Nemunas, Vilkija	PQ570396	−
	larva	FP593	*Neogobius fluviatilis*	River Nemunas, Vilkija	PQ570397	−
	larva	FP615	*Neogobius fluviatilis*	River Neris, Skirgiškės	PQ570398	−
	larva	FP646	*Neogobius fluviatilis*	River Neris, Skirgiškės	PQ570399	−
	larva	FP648	*Neogobius fluviatilis*	River Nemunas, Vilkija	PQ570400	−

*sequence of the 18S rDNA gene, **sequence of the ITS2 region
